# Patient-Matched 3-D-Printed Guides for the Insertion of Cervical Pedicle Screws in Tumor Surgery

**DOI:** 10.1155/2021/8135996

**Published:** 2021-11-28

**Authors:** C.-E. Heyde, G. Osterhoff, Spiegl UJA, A. Völker, N. H. von der Höh, J. S. Jarvers

**Affiliations:** Spine Division, Department of Orthopedics, Trauma and Plastic Surgery, University of Leipzig, Liebigstrasse 20, 04103 Leipzig, Germany

## Abstract

**Background:**

Pedicle screw fixation in the cervical spine provides biomechanical advantages compared to other stabilization techniques. However, pedicle screw insertion in this area is challenging due to the anatomical conditions with a high risk of breaching the small pedicles and violating the vertebral artery or neural structures. Today, several techniques to facilitate screw insertion and to make the procedure safer are used. 3-D-printed patient-matched guides based on a CT reconstruction are a helpful technique which allows to reduce operation time and to improve the safety of pedicle screw insertion at the cervical spine.

**Cases:**

3-D-printed patient-matched drill guides based on a CT scan with a 3-D reconstruction of the spine were used in two challenging cervical spine surgical tumor cases to facilitate the implantation of the pedicle screws. The screw position was controlled postoperatively by means of the routinely performed CT scan.

**Results:**

Postoperative imaging (conventional radiographs and CT scan) revealed the correct position of the pedicle screws. The time needed for screw insertion was short, and the need for intraoperative fluoroscopy could be reduced. There was no intra- or postoperative complication related to the pedicle screw implantation. Both tumors could be removed completely.

**Conclusion:**

These preliminary results show that 3-D-printed patient-specific guides are a promising tool to support and facilitate the implantation of cervical pedicle screws. The time needed for insertion is short, and intraoperative fluoroscopy time can be reduced. This technique allows for both a meticulous preoperative planning and a correct and therefore safe intraoperative positioning of cervical spine pedicle screws.

## 1. Introduction

Cervical pedicle screws are the most stable fixation technique at the posterior cervical spine. Due to the three-column fixation, biomechanical superiority compared to other screw techniques (lateral mass screws, lamina screw) could be proven [1–3]. However, the implantation of cervical pedicle screws remains a challenging procedure. This is related to the unique anatomical conditions of the cervical spine pedicles, particularly in the region from C3 to C6. The pedicles in this area are very thin with a large convergent angulation of the pedicle trajectory. Thus, there is a risk for pedicle violation and screw malpositioning associated with a potential loss of stability and an increased risk of injuring both the vertebral artery and the neural structures [1–4].

Hence, a meticulous preoperative planning, surgeon's expertise, and a thorough knowledge of the anatomy are crucial. Based on these prerequisites and an additional training program, some experienced surgeons recommend a free-hand screw insertion [3]. In contrast, the literature shows a higher complication rate compared to alternative screw techniques, especially in the mentioned region between C3 and C6 [1, 2, 4]. Therefore, different techniques to support and facilitate the implantation of cervical pedicle screws are used with the overall objective to improve the safety of the procedure. Various image-guided techniques and image-guided navigation techniques are reported in the literature. These techniques were able to prove higher accuracy of screw position compared to the free-hand technique [1, 2, 5].

In addition, first results of 3-D-printed drill guides to determine the right screw trajectory are available. Data from lumbar spine cases [6–8], from deformity cases [9–11], and first cases about pedicle screws in the upper and lower cervical spine [4, 12–14] are accessible.

The rationale behind this principle is to provide the surgeon with a 3-D-printed patient specific drill guide, which can be used intraoperatively without further imaging support and independently from changes in the position of the cervical spine. This guide is based on a preoperative CT scan with a 3-D reconstruction of the spine which allows to evaluate the best and safest screw trajectory.

## 2. Case Presentations

We represent the use of 3-D-printed patient-matched drill guides (MySpine, Medacta International SA, Castel San Pietro, CH) in two cases with a cervical spine tumor requiring a complete tumor resection and subsequent stabilization and fusion of the cervical spine. In both cases, an aggressive osteoblastoma that is characterized by aggressive growth and a high risk for local recurrence had to be removed completely. Meeting the standard, a meticulous diagnostic workup with conventional radiographs, CT-scan, MRI, PET, laboratory diagnostic, biopsy with reference pathology, and interdisciplinary decision-making in the tumor board was made. Interdisciplinary approach for resection, stabilization, and fusion as well as the postoperative therapeutic approach was planned.

**Figure 1:**
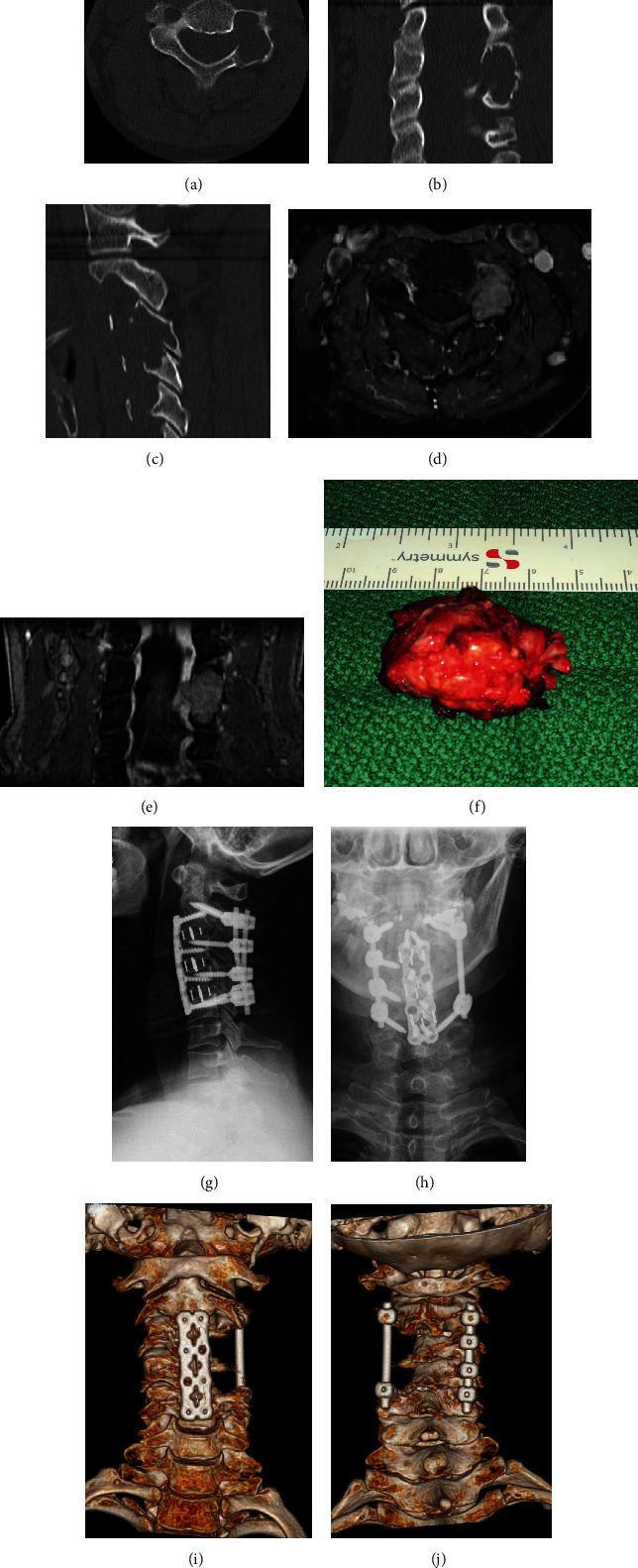
Case 1: aggressive osteoblastoma in C3/C4. (a–c) CT scan reconstructions of the cervical spine with a left-sided osteolysis in C3 and C4. (d, e) MRI shows the extent of the tumor and its close relation to the vertebral artery. (f) En bloc resected tumor. (g, h) Postoperative a.p. and lateral radiographs after resection of the tumor and combined posteroanterior stabilization. (i, j) CT scan 3-D reconstructions show the osseous defect after tumor resection.

*Case 1*: a 46-year-old female with refractory pain in the cervical spine, the head, and the upper arm at the left side for two years with rising intensity. She had a high need for pain medication. The clinical examination revealed a reduced range of motion of the cervical spine and a sensible radicular syndrome corresponding to the nerve roots C4-C6. The laboratory tests showed no hint for tumor or infection. Radiographs, CT scan, and MRI revealed a tumor at the left side C3-C4 in the cervical spine (Figure 1) without further pathologies in the complete tumor workup (PET-CT, laboratory tests). The biopsy and additional reference pathology yielded the result of an aggressive osteoblastoma (Enneking Type III). Hence, in the interdisciplinary tumor board, the decision for complete wide resection was made. Radiation was considered in dependence on the final pathology after tumor resection. An unremarkable occlusion test of the left vertebral artery was performed in order to simulate a potentially necessary ligation.

**Figure 2:**
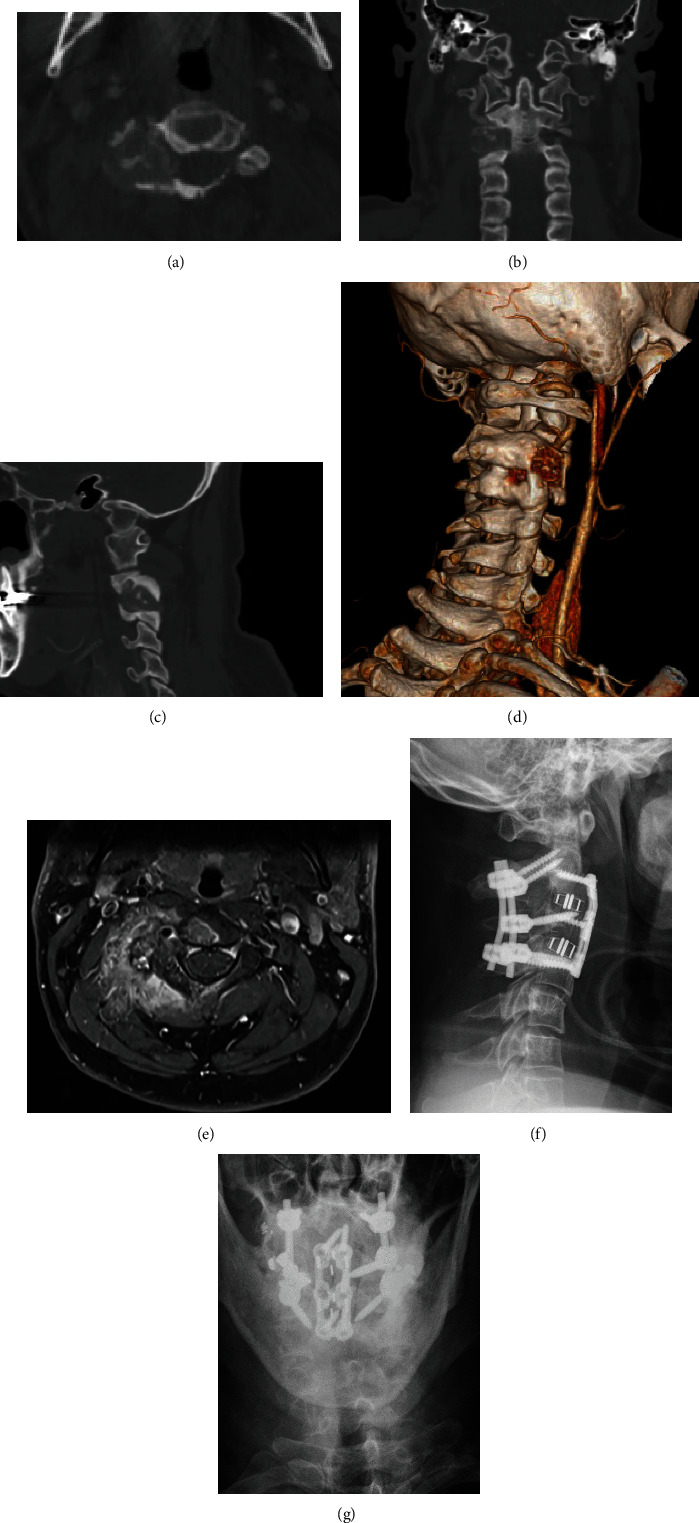
Case 2: aggressive osteoblastoma in C3. (a–d) CT scan reconstructions of the cervical spine with a right-sided lesion in C3. (e) MRI shows the soft-tissue component of the tumor. (f, g) Postoperative a.p. and lateral radiographs after resection of the tumor and combined posteroanterior stabilization.

*Case 2*: a 32-year-old patient with numbness in the fingers 3-5 and pain in the right shoulder and arm, particularly at night. Apart from a slightly reduced range of motion of the cervical spine and the mentioned sensory deficits, no noticeable clinical finding was observed. The radiographs, CT scan, and MRI revealed a tumor of C3 at the right side of the cervical spine (Figure 2). The further evaluation (PET, laboratory tests) showed no sign for an additional tumor localization or infection. The biopsy including the reference pathology confirmed the suspected aggressive osteoblastoma (Enneking Type III). A complete wide resection was recommended in the interdisciplinary tumor board. The decision about postoperative radiation was postponed because the final pathological finding after tumor resection should be included into the decision-making process. An uneventful occlusion test of the vertebral artery was performed.

### 2.1. Preoperative Planning and Template Manufacturing

For the planned pedicle screws, 3-D-printed patient-matched drill guides (MySpine, Medacta International SA, Castel San Pietro, CH) were planned and manufactured.

**Figure 3:**
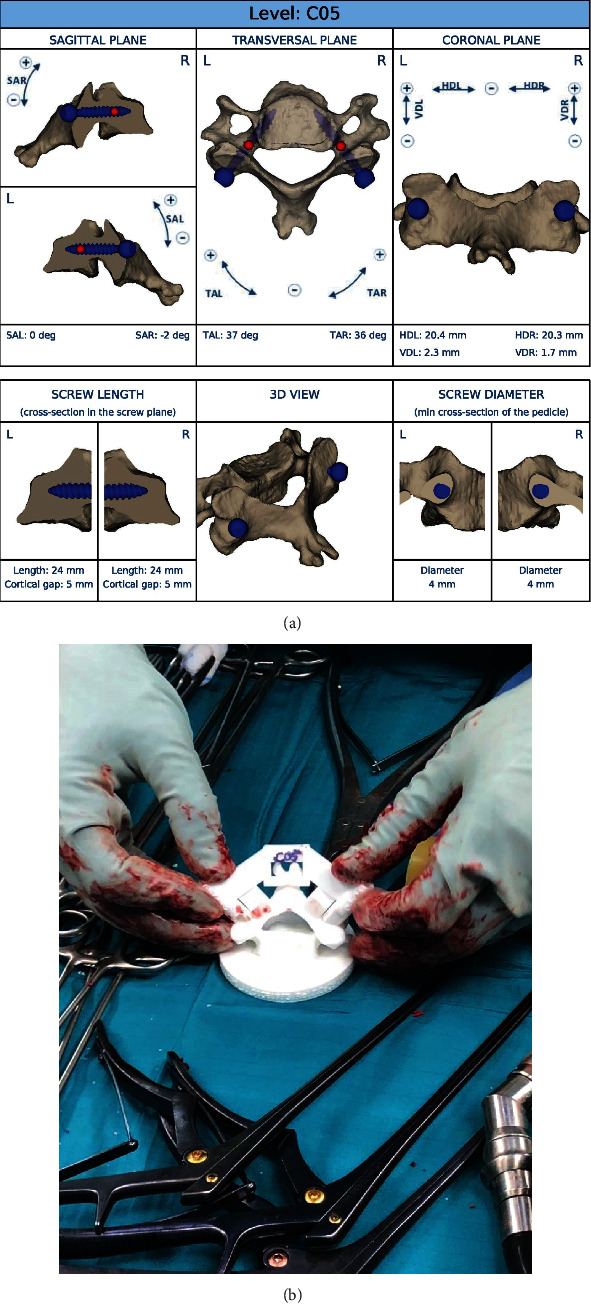
Drill guide templates. (a) Exemplary virtual presentation of the template provided by the manufacturer. This allows for meticulous planning of the entry point and the different screw parameters. (b) Shows the possibility to test the fit between the drill guide and the corresponding vertebra *ex situ*.

For this, a CT scan is required and a 3-D model of every single vertebra which is planned to be instrumented will be virtually reconstructed. Based on these data, the trajectory, the length, the diameter, and the entry point of every single screw can be planned individually. On a standardized platform provided by the company, a template with these proposals for the screw parameters is available for the surgeon (Figure 3(a)). All parameters can be validated and, if necessary, adapted by the surgeon. Not until the final template is released by the surgeon the process of manufacturing the individual drill guide as well as the model of the corresponding vertebral body will be started. The templates and the corresponding vertebra model will be delivered nonsterile. This allows the surgeon to test the fit of the template on the lamina of the respective vertebra before the surgery. During surgery, the sterile device can be used to get a better feeling for the proper placement of the template (Figure 3(b)).

### 2.2. Surgical Technique

En bloc tumor resection was performed with combined anterior and posterior approaches under IONM and partially under microscopic control in both cases.

During the posterior part of the surgical procedure (decompression, tumor liberation, tumor resection, and fusion), pedicle screws were inserted using the prepared 3-D-printed patient-matched drill guides. The posterior approach was performed as usual; however, a meticulous soft tissue removal from the bony structures was necessary to avoid a mismatch between the template and the posterior bony structures. After identification of the respective vertebra, the corresponding sterile template was placed on the lamina. The individual shape enables a perfect fit fixing all degrees of freedom. Once the template was placed stable and in a perfect fitting position, sleeves were used in order to adjust the diameter of the template to the diameter of the drill. Then, the pedicle hole was drilled. The first drill was let in place to achieve a greater stability during drilling of the hole on the other side. Afterwards, the drill on one side was removed and the intactness of the pedicle was confirmed with a ball-tip probe. After tapping the other side, the template was removed and the polyaxial screws were inserted in accordance with the preoperatively defined parameters. To improve the safety, lateral fluoroscopy can be used to control the trajectory during the drilling procedure. The template that is provided preoperatively allows the surgeon to compare the intraoperative trajectory of both the drill and the screws with the preoperatively planned trajectories in all desired planes.

*Case 1*: in this case, a complete anteroposterior en bloc tumor resection was possible under protection of the nerves and without affecting the vertebral artery. In order to realize a resection with a wide margin, a partial removal of the vertebral bodies C3 and C4 and the discs was necessary. Therefore, stabilization and fusion of C2 to C5 were performed with pedicle screws using the 3-D-printed patient-matched guides for posterior stabilization.

*Case 2*: in this case, an en bloc resection was possible via an anteroposterior approach. The tumor could be removed completely and stabilization and fusion of C2 to C4 were performed, using the above-described technique for the pedicle screws. During tumor dissection, a violation of the vertebral artery took place caused by their close course directly to the tumor; therefore, an occlusion of the artery was necessary.

**Figure 4:**
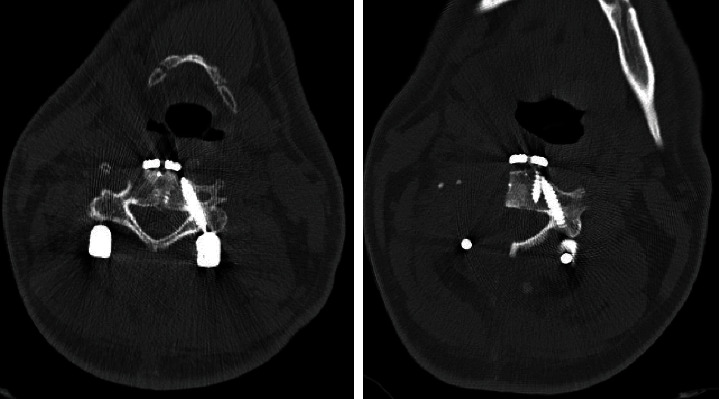
Precision of screw positioning. Axial reconstructions of the postoperative CT scan (case 2) to evaluate the correct placement of the pedicle screws. In both examples, the correct position of the screws could be confirmed.

Postoperative screw position was evaluated by a routinely postoperative performed CT scan (Figure 4). The screw placement accuracy was graded based on the degree of perforation of the pedicle (grade 0 (contained), grade 1 (exposure), grade 2 (perforation), and grade 3 (penetration)) as previously described by Wu et al. [4].

### 2.3. Outcome

In these two presented cases, the cervical pedicle screws were inserted using the MySpine technology, 3-D-printed patient-matched drill guides.

Time for insertion of all screws (from placing the template first time to final screw insertion of the last screw) was 8 minutes (6 screws) in the first and 10 minutes (5 screws) in the second case. Fluoroscopy was performed during drilling only in the lateral plane to confirm the trajectory compared to the preoperative planning. At the end of the procedure, an a.p. view was performed in addition.

**Figure 5:**
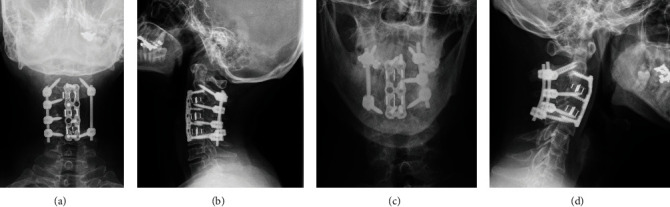
Follow-up. (a, b) a.p. and lateral radiographs of the cervical spine of case 1 at 12 months postoperatively. (c, d) a.p. and lateral radiographs of the cervical spine of case 2 at 4 months postoperatively.

*Case 1*: postoperatively, the patient showed new dysesthesia corresponding to the C3 and C4 nerve roots and slightly weakened shoulder abduction at the operated side. The wound healing was uneventful, and the neurological impairment recovered completely within 4 months. R-0 resection was confirmed by histopathological examination. Postoperative radiation according to the tumor board decision based on the confirmed diagnosis of an aggressive osteoblastoma was initiated. At 1-year follow-up, the patient reported about minimal pain but a markedly reduced range of motion. There was no neurological deficit, and imaging (radiographs and MRI) showed neither implant loosening nor local recurrence (Figures 5(a) and 5(b)).

*Case 2*: in accordance with the preoperative occlusion test, there was no neurological deficit caused by the closure of the vertebral artery. A postoperative arteriography revealed a good blood supply. Wound healing was uneventful and there was no neurological deficit. R-0 resection could be confirmed by histopathological examination. In the follow-up examination four months postoperatively, the patient reported about minimal pain and a reduced range of motion of the cervical spine without any neurological problem. In the meantime, radiation was performed due to the confirmed diagnosis of an aggressive osteoblastoma. The radiographs at follow-up revealed unchanged and unremarkable findings (Figures 5(c) and 5(d)).

Screw position was evaluated by the postoperative CT scan. This showed a correct positioning of all screws. Eight pedicle screws showed perfect accuracy with no penetration of the pedicle (grade 0), and pedicle exposure (grade 1) was observed in three cases.

## 3. Discussion

Cervical pedicle screws are biomechanically more stable than alternative screw fixation techniques [1–3]. Due to the challenging anatomical conditions, the risk of relevant complications with the free-hand technique is high [1, 2]. Image-guided navigation techniques were able to prove an improvement regarding the safety of cervical pedicle screw insertion. A higher accuracy in screw placement could be shown [1, 2, 5].

Alternatively, 3-D-printed patient-matched guides can be used to facilitate the procedure and allow for a quick and safe screw insertion of cervical pedicle screws. The feasibility of the technique of 3-D-printed patient-specific guides for screw insertion could be shown for different indications in case of lumbar diseases and spinal deformities [6–11]. In addition, first reports about their use in the upper and lower cervical spine are available [4, 12–14]. All these reports could prove the safety of the procedure regarding a correct screw placement. Kashyap et al. [13] introduced a technique where the whole process of planning and manufacturing of the guides must be realized by the surgeon itself. From our point of view, the opportunity to us a template provided by the manufacturer with a proposal for the screw position and parameters which can be adapted seems to be a great advantage. In addition, the supply of both the 3-D-model of the respective vertebra and the corresponding template allowing both preoperative control and intraoperative matching is helpful. One of the major advantages of this technique is its application regardless of patient positioning. There is no need for additional intraoperative matching. Thus, fluoroscopy time can be reduced, and the procedure is not time-consuming.

However, this technique is only appropriate for elective cases. The process of planning and manufacturing is time-consuming [12]. In addition, a wide exposure is necessary due to the required angulation of the screw trajectories. New developments which might allow percutaneous screw insertion could reduce the need of wide exposure and are desirable. The two cases demonstrated in this article were of relatively young age and both showed only very little degenerative changes to the posterior aspects of their cervical spines. In case of severe degenerative changes, fitting of the guide might be more difficult and require more time.

## 4. Conclusions and Clinical Message

In these two presented cases, we were able to remove aggressive osteoblastoma Type III of the cervical spine completely in accordance with the recommendations of the literature.

These procedures in a challenging anatomical area with the need of en bloc resection and a combined anterior and posterior approach are time-consuming. In addition, if a relevant weakening of the structures is necessary, stabilization and fusion are indicated. Therefore, every tool that may reduce surgery time and improves the safety of the procedure is helpful. The presented technique meets all these criteria.

## Data Availability

Raw data can be provided by the corresponding author upon request.

## Abbreviations

c: Cervical
CT: Computed tomography
3-D: Three-dimensional
IONM: Intraoperative neuromonitoring
MRI: Magnetic resonance imaging
PET: Positron emission tomography.
